# Hemolysis induced cross-matching difficulty with intravenous immunoglobulin: a case report

**DOI:** 10.1186/s13256-018-1774-0

**Published:** 2018-09-03

**Authors:** Achyut Sharma, Diptesh Aryal

**Affiliations:** Department of Critical Care, Nepal Mediciti Hospital, Nakhkhu Patan, Karyabinayak, 44600 Nepal

**Keywords:** IVIG, Guillain–Barré syndrome, Cross-matching, Hemolysis with IVIG, GBS

## Abstract

**Background:**

Intravenous immunoglobulin is one of the most common modalities of treatment for Guillain–Barré syndrome. Although minor complications are easily preventable with pre-medications, rare complications like hemolysis occur at unexpected times and carry risks of repeated transfusions. A complication like difficulties in cross-matching blood is an uncommon event and is often not anticipated. We present one such rare case.

**Case presentation:**

A 56-year-old man of Asian origin had presented to our hospital with rapidly progressive weakness of bilateral upper and lower limbs over 4 days. Guillain–Barré syndrome was diagnosed by nerve conduction velocity testing and lumbar puncture examination. On the third day of admission in hospital he was intubated because of respiratory failure. Intravenous immunoglobulin at 0.4 mg/kg per day for 5 days was planned and started. Our patient was scheduled for tracheostomy on a routine basis anticipating prolonged requirement of ventilator support. As the blood was being arranged, the blood bank facilities informed us about difficulties in cross-matching of the blood. Repeated samples and attempts at cross-matching were futile. After reviewing the available literature and diagnosing a case of hemolysis, relevant tests were performed and they were positive.

**Conclusions:**

Anti-A and anti-B antibody present in intravenous immunoglobulin preparations sensitize the red blood cells to hemolysis and this occurrence is often incriminated as a cause of cross-matching and sometimes blood grouping difficulty. Although a high dose of intravenous immunoglobulin or repeated courses are often cited as reasons for hemolysis, individual variability in responses is common and it is not surprising to see one like we had in our case.

## Background

Intravenous immunoglobulin (IVIG) [1] and plasmapheresis [2] are the two most common treatment approaches to Guillain–Barré syndrome (GBS). The individual superiority of each of these techniques is variable among different clinical trials [3–5]. Thus, both of these modalities are favored over each other based on the local clinical practice, responses to previous therapies, and the treating physician’s expertise. The treatment of GBS with IVIG has the advantages of being relatively cost-effective and easy to administer. However, unusual complications and reactions, although uncommon, occur at times and are ambiguous enough to affect management. We present one such infrequently reported problem of difficulties in cross-matching the blood of a patient who underwent IVIG treatment and present a cautionary note to avert the serious consequences that may follow.

## Case presentation

A 56-year-old man of Asian origin was in his usual state of health until 4 days prior to presentation in our emergency room (ER); he had complaints of a rapidly progressing weakness of bilateral upper and lower limbs immediately prior to which he had a gastrointestinal upset. The weakness started from his lower limbs and gradually involved bilateral upper limbs in a similar glove and stocking fashion. He, however, did not have any sensory deficits and at the initial presentation in our hospital he had no respiratory and ocular muscle involvement. Consciousness and orientation were intact and he did not have significant hemodynamic instability. There was no significant medical or surgical condition requiring long-term hospitalization or medication use in the past. A government officer by profession, our patient had an active lifestyle and had no history suggestive of substance abuse or accidental or intentional poisoning. He lived with his wife and had two sons; one of his sons was living with him to support him and the elder son lived abroad but had been on good terms with the family. Our patient did not take any regular medications apart from the hypoglycemic agent metformin 500 mg administered orally twice daily. He did not smoke tobacco or consume alcohol regularly. During his initial presentation in our intensive care unit (ICU), he was conscious yet unable to speak properly. His vital signs were blood pressure (BP) 120/65 mmHg with no inotropic support, heart rate (HR) 102/minute regular, respiratory rate (RR) 26/minute regular, and he had no fever on admission.

Our initial assessment led to a provisional diagnosis of GBS and immediate supportive tests were performed. A nerve conduction velocity test showed findings of motor axonal and demyelination neuropathy. A lumbar puncture done on the sixth day of the development of symptoms showed evidence of albuminocytologic dissociation with total counts (TC) of five cells/cc, which were all lymphocytes and CSF protein of 81 mg/dl (Table 1). On the sixth day of the development of symptoms and third day of admission in ICU, he had complaints of difficulty in breathing with gradual decline in saturation with pulse oximeter reading of oxygen saturation (SpO_2_) to < 85% at fraction of inspired oxygen concentration (FiO_2_) of > 80%, and hypercapnia with partial pressure of carbon dioxide in arterial blood (PaCO_2_) of 86 mmHg. He was immediately intubated and kept on mechanical ventilator support.

**Table 1:** Significant investigations during course of treatment

Parameters/Date	Day 2	Day 4	Day 5	Day 7	Day 8	Day 9	Day 23	Day 34	Day 55	Day 60
Hb (g/dl)	15.3	14.2	13.6	12.9	11.3	11.6		10.5		
Platelets (cells/mm^3^)	169,000	160,000	145,000	141,000	150,000	146,000		170,000	345,000	
Total leukocyte counts (cells/mm^3^)	11,540	11,480	8930	8850	8380	7460		8560	14,770	
Hb									9.9	
CSF protein (mg/dl)	81						88			
CSF glucose (mg/dl)	160						160			
CSF total counts	5.00 cells/mm^3^; all lymphocytes						5; all lymphocytes			
Total serum protein (g/dl)	7.9			8.7		7.5				
Albumin (g/dl)	3.7			3.5		3.0				
Bilirubin total (mg/dl)	1			0.5		0.9				
Bilirubin (conjugated)	0.1			0.2		0.2			0.2	0.1
Bilirubin unconjugated	0.8			0.3		0.4			0.4	0.4
ALT	82			51		197			347	413
ALP	101			85		116			248	378
TFT	Normal									
Na+	140	140	148	148	148	147			146	
K+	4.2	4.4	3.9	4.0	3.9	4.2			4.3	
Urea	78	75	65	45	52	55			85	
Creatinine	0.8	1.0	0.9	0.9	1.0	1.1			0.6	
Vitamin B12 (pg/ml)				863						
Calcium (mg/dl)				8						
Phosphorus (mg/dl)				4.2						
Sputum C/S							*Klebsiella*, Pneumonia, *Pseudomonas aeruginosa*			
Urine C/S							No growth			
Blood C/S							No growth			
PT-INR								13.7 seconds/1.37 aPTT 23 seconds		
Urine R/M				Normal						
Parameters/Date	Day 72	Day 76	Day 83	Day 90	Day 101	Day 109	Day 112	Day 117	Day 118	Day 122
Hb (g/dl)		8.3			9.3		9.8		9.6	
Platelets (cells/mm^3^)		256,000			245,000		253,000		234,000	
Total leukocyte counts (cells/mm^3^)		13,440			12,600		8670			
CSF protein (mg/dl)										
CSF glucose (mg/dl)										
CSF total counts										
Total serum protein (g/dl)	5.6		6.8					6.9		
Albumin (g/dl)	2.4		3.0			3.6	3.7	3.5		
Bilirubin total (mg/dl)	0.6		0.4			0.4	0.3			
Bilirubin (conjugated)	0.4		0.1			0.1	0.1			
Bilirubin unconjugated	0.2		0.2			0.5	0.2			
ALT	278		208			44	54			
ALP	207		198			26	55			
TFT										
Na+	151		142						143	
K+	3.7		3.1						3.7	
Mg+	2.7								2.2	
Urea	102							54	55	
Creatinine	1.1							1.0	1.1	
Vitamin B12 (pg/ml)										
Calcium (mg/dl)								8.0		
Phosphorus (mg/dl)								4.6		
Sputum C/S		No growth			*Acinetobacter* species					
Urine C/S		No growth								
Blood C/S		No growth								
PT-INR									11.4/1.2	
Iron profile	Iron, 18 mcg/dl; TIBC, 207 micmol/L; ferritin, 765 ng/ml; % sat, 8.69									
HbA1c				6.3%						
Urine R/M					Normal					

*ALP* alkaline phosphatase, *ALT* alanine transaminase, *aPTT* activated partial thromboplastin time, *C/S* culture and sensitivity, *CSF* cerebrospinal fluid, *Hb* hemoglobin, *HbA1c* glycated hemoglobin, *PT-INR* prothrombin time-international normalized ratio, *R/M* complete examination, *sat* saturation, *TFT* thyroid function test, *TIBC* total iron-binding capacity

Discussion was held with his relatives regarding available treatment options. A plan to initiate IVIG was made and started at 0.4 mg/kg per day for 5 days. The administration of IVIG was not associated with any significant complications. He did not, however, show major signs of recovery from respiratory weakness and was continuously kept on assist-control mode of ventilation with intermittent spontaneous breathing trials. At this time, he had occasional blood-tinged secretion in the subglottic suction and during intermittent endotracheal suctioning. A detailed coagulation profile did not show significant abnormalities. On the ninth day of admission in ICU, a plan for tracheostomy was made anticipating prolonged need for mechanical ventilation and as a part of routine pre-anesthetic preparation, a unit of group-specific (A +ve) blood was asked to be arranged. However, we were then notified by the blood bank that they had problems with cross-matching of the blood. A repeated blood sample of our patient was sent which also had a similar problem of inability to cross-match the blood. A literature search for the possible causes of such an occurrence was made but we only had a few reports of such problems. With a provisional diagnosis of significant hemolysis leading to cross-matching difficulties, further tests were sent (Table 1). An arrangement of O negative blood was made as a reserve and a tracheostomy was performed with no major problems.

His stay in ICU was then complicated with hospital-acquired chest infections for which he received antibiotics based on organisms’ susceptibility. Liver function tests (LFTs) which were initially deranged subsequently normalized after gradual stabilization of his condition and de-escalation of drugs. Serological tests which included quantitative HIV, hepatitis B surface antigen (HBsAg), and anti- hepatitis C virus (HCV) antibody were negative. He was subsequently moved out of ICU on the 19th day with tracheostomy *in situ* and on portable bilevel positive airway pressure (BIPAP) support. Three days after being moved to a ward, he was brought back to ICU for sudden-onset dyspnea and tachypnea. He had coarse crepitations more on the anterior aspect of bilateral chest and slight decrease in breath sounds on bilateral basal regions. He did not, however, have fever or changes in vital signs and his consciousness was intact. He was managed conservatively with chest physiotherapy, deep breathing exercises, regular tracheostomy care, and suctioning of secretions from lungs. He recuperated in 2 days and was moved back to a ward where he slowly recovered from his weakness. No other untoward events occurred during this period of approximately 4 months. He is being planned for discharge to the care of a nursing home and the prognosis of the disease has been well explained to his relatives.

## Discussion

Although minor complications like changes in hemodynamics, anaphylaxis, and renal dysfunction are common occurrences with IVIG and have been frequently reported, there are very few reported cases in the literature on difficulties in cross-matching blood; thus, it may come as a surprise to any unsuspecting critical care physician. This case report may serve as a cautionary note to all of us.

The use of immunoglobulins as treatment for GBS dates back to 1952 when the first successful use of immunoglobulins was done by Bruton. However, the successful use of immunoglobulin in its current intravenous form was only possible in 1981 [6]. IVIG is also used for various other conditions like idiopathic thrombocytopenic purpura, primary immune deficiency, myeloma, chronic lymphocytic leukemia, and Kawasaki disease among others [7]. Barring a contraindication of previous anaphylaxis, clinical experience with IVIG has generally been favorable for GBS. Common complications like fall in BP and anaphylaxis requiring oxygen and adrenaline during the starting of IVIG are well established and pre-medications in the form of antihistamines and antipyretics are used before the first dose of each day [8]. Renal dysfunction and aseptic meningitis are less frequent, easily recognizable, and treatable if recognized on time with no serious sequelae if properly and timely managed. Yet another less frequently reported problem is that of hemolysis; its occurrence with IVIG treatment is cited to be around 0.1 to 1% [9].

IVIG is prepared from a plasma pool of at least 1000 donors and should contain at least 90% intact IgG and those molecules must be able to maintain biological activity such as complement activation. These preparations (a total of 25 different types available worldwide) should be free from preservatives, proteases, kinins, and infectious agents [10]. As these preparations are made from pooled donors preferably from patients with blood group O, there are chances of the creeping in of anti-A, anti-B, and sometimes anti-D or other red blood cells (RBCs) antibodies in the product during the preparation. The problem that arises because of these constituents of IVIG is hemolysis, although it is usually reported with very high dose and in patients with blood group A, AB, or B [11]. Such conditions are also reported to have occurred more frequently in persons with reduced bone marrow reserve or post hemopoietic stem cell transplantation [12].

In our case we observed several commonly encountered complications in the course of treatment of GBS, such as respiratory complications requiring intubation and later tracheostomy, common electrolyte imbalances like hypokalemia and hypocalcemia, a relatively uncommon complication of hypernatremia (Table 1) which is also reported very infrequently, and a common gastrointestinal problem like constipation. Patients with GBS admitted in critical care units are very sick with a multitude of problems. Thus, it is very easy to sometimes overlook conditions like hemolysis in an asymptomatic case or when we are too busy with several other more serious complications. In this example too, our patient had signs of autonomic dysfunction with labile BP reaching maximum up to 212 mmHg, he had fluctuating HR, and developed hospital-acquired pneumonia. A possible consequence of such a slip up is bleeding from orifices, intravenous cannula sites, endotracheal suction, and subglottic suction.

An uncommon and infrequently encountered yet equally concerning consequence of hemolysis is a problem with cross-matching. When we imagine a patient who has falling hematocrit because of hemolysis and requires transfusion of group-specific blood, but has a problem getting a transfusion of group-specific blood due to trouble with the cross-matching process, the consequence of non-transfusion is often death due to bleeding. The presence of anti-A, anti-B, and sometimes anti-D antibodies in the recipient blood (sent for cross-matching) has often sensitized the red cells resulting in transient hemolytic anemia and cross-matching difficulties. Fortunately, since we had had administered a relatively lower dose of IVIG, there was no trouble in obtaining the ABO and Rh group of our patient. However, it is reported that a person receiving large or repeated doses of IVIG often has difficulties with blood grouping. A pre-infusion ABO blood group determination is often advised by some manufacturers. Given the problem that we faced, that does not seem to be illogical.

Yet another interesting finding that we noticed from this case is the importance of knowing a pre-infusion blood group and anticipation of hemolysis. As these IVIG solutions contain anti-A and anti-B antibodies, an O group person is less liable to be affected by hemolysis than a group A or AB person is. Although previous literature and manufacturer leaflets have suggested that serious forms of hemolysis only occur with a very high dose or repeated courses of IVIG treatment, we found that given the susceptibility of the person, he or she is liable to have hemolysis even with low dose treatment of IVIG. Thus, individual susceptibility to hemolysis plays a significant role [11]. We may, however, surmise that our anecdotal experience may require further validation.

Thus, as a form of preventive measure and at least for the purpose of early diagnosis of hemolysis, we suggest a few things as listed below to be considered when using IVIG, although these are just suggestions made from our observation and need to be scrutinized and compared with other guidelines from regulatory bodies.

1. Maintaining a protocol-based administration of IVIG for all patients with no ambiguous words.
2. A pre-infusion ABO grouping is a logical idea to follow.
3. A baseline LFT, renal function test (RFT), coagulation profile, hematocrit level, and hemoglobin level can be used for comparison if any changes are suspected.
4. Any symptoms like melena, bleeding from orifices, bleeding in endotracheal tube (ETT) suction, sub-glottic suction, and development of jaundice may be the harbingers of significant hemolysis; thus, there will be a need to do an immediate work-up for the patient with laboratory markers of hemolysis such as haptoglobin, lactate dehydrogenase, bilirubin total and direct, alkaline phosphatase, and so on.
5. In a case of severe bleeding leading to precipitous fall in hematocrit and in condition, when there is cross-matching difficulty, an O-negative blood transfusion can be lifesaving and it needs to be immediately arranged.

## Conclusions

With the ever growing critical care management of GBS with IVIG, it is not uncommon to encounter a variety of complications of which some are common and some less frequent. Although sufficient anticipation, preparedness, and expertise are available in the management of routine complications of IVIG treatment, atypical adverse reactions often pose diagnostic as well as management ambiguities like the one of cross-matching difficulty that we faced in our case. The lesson we learned from this case report is that it is important for all of us to be prepared for even the least observed problems in order to holistically manage a case of GBS.

## Abbreviations

BIPAP: Bilevel positive airway pressure
BP: Blood pressure
ER: Emergency room
ETT: Endotracheal tube
FiO_2_: Fraction of inspired oxygen concentration
GBS: Guillain–Barré syndrome
HBsAg: Hepatitis B surface antigen
HCV: Hepatitis C virus
HR: Heart rate
ICU: Intensive care unit
IVIG: Intravenous immunoglobulin
LFT: Liver function test
PaCO_2_: Partial pressure of carbon dioxide in arterial blood
RBCs: Red blood cells
RFT: Renal function test
RR: Respiratory rate
SpO_2_: Pulse oximeter reading of oxygen saturation
TC: Total counts
